# The complete chloroplast genome of *Sinomenium acutum* (Menispermaceae)

**DOI:** 10.1080/23802359.2020.1797570

**Published:** 2020-07-25

**Authors:** Jinhyuk Kim, Junki Lee, Sanghee Um, Sunseong Choi, Hyojin Kim, Hyang Sook Chun, Gyoungju Nah

**Affiliations:** a Genome Analysis Center at National Instrumentation Center for Environmental Management, Seoul National University, Seoul, Korea; b Department of Food Science and Technology, Chung-Ang University, Ahnsung, Korea

**Keywords:** *Sinomenium acutum*, complete chloroplast genome, next generation sequencing, phylogenetic tree

## Abstract

We generated the complete chloroplast genome sequence of *Sinomenium acutum*, a species of the Menispermaceae family, and characterized from the *de novo* assembly of Illumina HiSeq paired-end sequencing data. The total length of the chloroplast genome of *S. acutum* was 162,787 bp with a large single-copy (LSC) region of 91,430 bp, a small single-copy (SSC) region of 21,245 bp, and a pair of identical inverted repeat regions (IRs) of 25,056 bp. The total of 131 genes were annotated in the chloroplast genome of *Sinomenium acutum*, including 85 protein-coding genes, 38 transfer RNA (tRNA) genes, and 8 ribosomal RNA (rRNA) genes. The phylogenetic analysis of *S. acutum* with 18 related species revealed the closest taxonomical relationship with *Menispermum dauricum* in the *Menispermaceae* family.

*Sinomenium acutum,* also known as Chinese moonseed, is a species belonging to the Menispermaceae family which is native to east Asia (Ortiz et al. 2007). The root of *Sinomenium acutum* has been used as medicinal material in China and Korea due to its treatment effect on anti-inflammatory (Zhao et al. 2015; Kim et al. 2018) and rheumatic disease (Lyu et al. 2018). Still many medicinal plants, including *Sinomenium acutum,* are lack of DNA barcode markers and we generated and the complete chloroplast genome sequence of *S. acutum*, using next generation sequencing for future resource for marker development. In addition, the sequence of chloroplast genome of *Sinomenium acutum* would assist future study on genetic diversity and conservation of Menispermaceae speceis.

The plant sample of *Sinomenium acutum* was maintained and collected from Medicinal Herb Garden, College of Pharmacy, Seoul National University (http://snuherb.snu.ac.kr/) in Goyang, Korea (37°42′44.9″N, 126°49′08.0″E). Total genomic DNA from leaves was used to construct the genomic library for Illumina paired-end (PE) sequencing and also deposited in National Institute of Biological Resources (42 Hwangyeong-ro, Seo-gu, Incheon, 22689, Korea) with collection number of NIBRGR0000622205. The high quality HiSeq reads (>Q30) were assembled by CLC Genomics Workbench (ver. 10.0.1, CLC QIAGEN), followed by manual curation through PE reads mapping (Kim et al. 2015). Annotation of the complete chloroplast genome was performed with GeSeq and manual corrections (Tillich et al. 2017). The complete chloroplast genome sequence of *S. acutum* was submitted to GenBank with the accession number of MN626719.

Total length of complete chloroplast genome of *S. acutum* was 162,787 bp with 37.8% of G + C content, comprising a large single copy (LSC) region of 91,430 bp, a small single copy (SSC) region of 21,245 bp, and a pair of inverted repeat (IRa and IRb) regions of 25,056 bp. The genome contained 131 genes including 85 protein-coding genes, 38 tRNA genes, and 8 rRNA genes.

To validate the phylogenetic relationship, the complete chloroplast genome sequences of *S. acutum* and those from 18 related species were aligned using MAFFT (ver. 7.271) (Katoh et al. 2002), followed by phylogenetic tree construction obtained from a Maximum Likelihood (ML) analysis with 1,000 bootstraps using MEGA 7.0 (Kumar et al. 2016). The phylogenetic tree exhibited the close relationship of *S. acutum* with *Menispermum dauricum* in the family of Menispermaceae (Figure 1).

**Figure 1.**
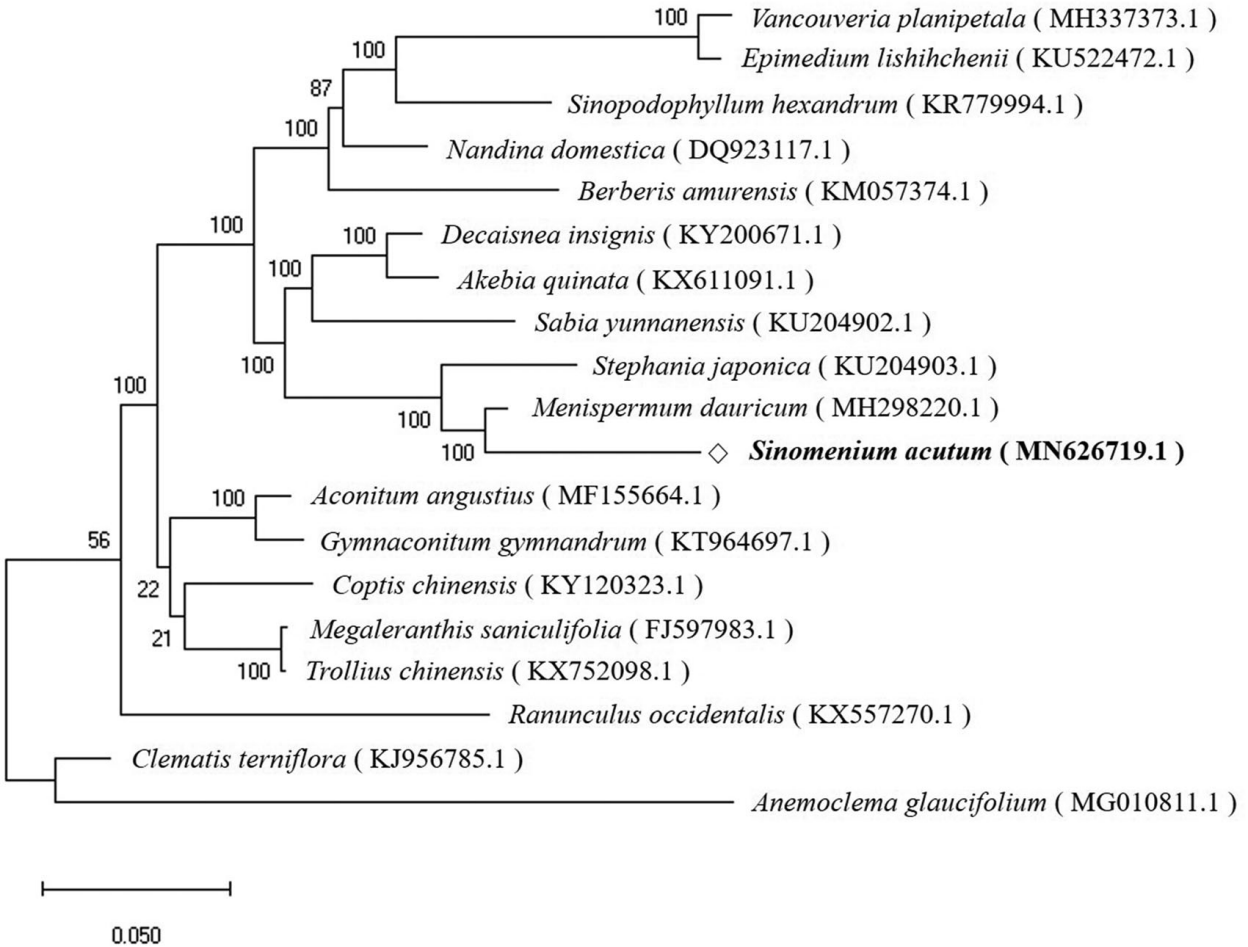
The phylogenetic tree was constructed using total chloroplast genome sequences of 18 species using Maximum Likelihood (ML) method with bootstrap values from 1,000 replicates.

## Data Availability

The data that support the findings of this study are openly available in GenBank of NCBI at https://www.ncbi.nlm.nih.gov, reference number of MN626719.
